# Robust zero-watermarking for color images using hybrid deep learning models and encryption

**DOI:** 10.1038/s41598-025-09290-7

**Published:** 2025-08-07

**Authors:** Hager A. Gharib, Noha M. M. Abdelnapi, Khalid M. Hosny

**Affiliations:** 1 Department of Computer Science, Faculty of Computers and Information, Suez University, P.O. BOX: 43221, Suez, Egypt; 2 Department of Information Technology, Faculty of Computers and Information, Zagazig University, P.O.BOX:44519, Zagazig, Egypt

**Keywords:** Zero-watermarking, Local binary patterns, VGG19, DWT, DCT, Chaotic encryption, Copyright protection, Robustness against attacks, Multimedia security, Neural networks, Engineering, Mathematics and computing

## Abstract

Reliable zero-watermarking is a distortion-free approach to copyright protection, which has been a primary focus of digital watermarking research. Traditional zero-watermarking techniques often struggle to maintain resilience against geometric and signal processing attacks while ensuring high security and imperceptibility. Many existing methods fail to extract stable and distinguishable features, making them vulnerable to image distortions such as compression, filtering, and geometric transformations. This paper presents a robust zero-watermarking technique for color images, combining Local Binary Patterns (LBP) with deep features extracted from the CONV5-4 layer of the VGG19 neural network to overcome these limitations. Frequent domain transformations, utilizing the Discrete Wavelet Transform (DWT) and Discrete Cosine Transform (DCT), enhance feature representation and improve resilience. Furthermore, a chaotic encryption scheme based on the Lorenz system and the Logistic map is used to scramble the feature matrix and watermark, thereby ensuring increased security. The zero watermark is generated through an XOR operation, facilitating imperceptible and secure ownership verification. Experimental results show that the proposed method is highly resilient to various attacks, including scaling, noise, filtering, compression, and rotation. The extracted watermark maintains a low Bit Error Rate (BER) and a high Normalized Cross-Correlation (NCC). At the same time, the Peak Signal-to-Noise Ratio (PSNR) of attacked images remains optimal. Specifically, the BER values of the extracted watermarks were below 0.0022, and the NCC values were above 0.9959. In contrast, the average PSNR values of the attacked images reached 34.0692 dB, demonstrating the method’s superior robustness and visual quality. Compared to existing zero-watermarking algorithms, the proposed method shows superior robustness and security, making it highly effective for multimedia copyright protection.

## Introduction

The widespread use of computers, the internet, and multimedia has enabled the global sharing of digital data. However, the ease of access to image processing tools has increased the risk of unauthorized copying and modification, raising concerns about intellectual property protection and data integrity.

Various information security techniques have been proposed to address copyright protection issues. These techniques are broadly categorized into cryptography and information hiding methods. Cryptography transforms messages into secure formats accessible only to authorized individuals. However, once decrypted, the message becomes vulnerable to potential misuse. Additionally, cryptographic methods are often more computationally complex than information hiding techniques. In contrast, information hiding approaches such as watermarking and steganography offer alternatives that overcome some of the complexity and limitations of cryptography^1^.

The term steganography is derived from the Greek words steganos (meaning “covered”) and graphein (meaning “writing”), which together form the phrase “hidden writing.” In steganography, hidden information is imperceptible; only the intended recipient knows the concealed data. However, a key limitation of steganography is that unintended parties find hidden messages difficult to detect or retrieve, making it less practical for specific multimedia applications.

The primary distinction between steganography and watermarking lies in the method of embedding. In steganography, the cover object and the hidden message are generally unrelated, whereas in watermarking, the watermark and the host media may be related or unrelated. Moreover, watermarking techniques can be visible or invisible, while steganography is inherently invisible^1^.

Digital image watermarking effectively ensures owner identification, privacy protection^2^, content authentication^3^, and digital image validity^4–7^. Traditional methods embed watermark information into images for protection^8–12^, but they have limitations. These methods often distort images, compromising data integrity, which is problematic for military imaging, medical diagnosis, and artwork scanning applications. Moreover, balancing robustness and imperceptibility remains a challenge^13^.

Watermarking techniques are generally categorized based on the embedding domain into spatial domain and frequency domain techniques. Spatial domain methods directly modify pixel values, which makes them simpler but more vulnerable to attacks. Frequency domain methods, such as those based on the Discrete Wavelet Transform (DWT) and the Discrete Cosine Transform (DCT), embed watermarks in transformed coefficients, thereby improving robustness against common image-processing attacks^14^.

Deep learning, a branch of machine learning, is a significant area of artificial intelligence research that utilizes neural networks to analyze vast datasets^15^. It enhances watermarking by extracting visual attributes and adaptively embedding them^16^. Convolutional Neural Networks (CNN) play a key role in this process, utilizing convolution and pooling layers to capture essential features^17^.

Zero-watermarking technology enhances copyright protection for digital multimedia, preserving visual quality, particularly for images. Unlike traditional watermarking, zero-watermarking^18–23^ associates the watermark sequence with the image without embedding it, thereby ensuring the integrity of the watermark. Ownership is verified through a zero watermark, transmitted securely over public channels, relying on intrinsic features and a master share.

Zero-watermarking approaches are classified into four categories based on significant image features^23,24^: CNN-based features^25,26^, frequency-domain features, spatial-domain features^14^, and moment-based features^27^. In CNN-based methods, deep feature maps are combined to extract image features from the layers of a convolutional neural network (CNN)^25,26^. Frequency-domain methods use transformed features but struggle with rotational and scaling invariance^14^. Spatial-domain methods directly derive features but are highly sensitive to geometric and image-processing attacks^14^. In comparison, Moment-based approaches leverage invariants for feature extraction^27,28^.

The zero-watermarking technique aims to produce a watermark without altering the original data^29^. It extracts robust features from the host image, converts them into numerical values, and combines them to form the zero-watermark, which is then encrypted with copyright data^30^. Pang et al.^31^ introduced a blind watermarking method for protecting open-source datasets using a GAN-based deep learning model. This approach applies invisible watermarks to dataset images and utilizes UNet with GAN for watermark embedding. The watermark is extracted for verification if a model is suspected of improper training. Thanh et al.^32^ proposed a resilient zero-watermarking technique that employs QR decomposition and a visual map, utilizing permutation attributes to enhance resilience and minimize computational costs. Daoui et al.^33^ suggested a zero-watermarking strategy integrated with robust image encryption to strengthen security during image sharing over the internet. Ge et al.^16^ proposed a DNN-based watermarking method for document images, utilizing an encoder-decoder framework for embedding and extraction. A noise layer simulates various attacks, while a text-sensitive loss mechanism limits changes to character embeddings. Despite achieving adequate PSNR and SSIM, the method suffers from poor visual quality due to the visible background in the document image. They introduced an embedding strength adjustment technique to enhance image quality while maintaining extraction accuracy, addressing this issue. Shao et al.^34^ developed a resilient double-zero watermarking technique to simultaneously safeguard the copyrights of two images. Han et al.^25^ proposed a resilient zero-watermarking method leveraging the VGG19 deep convolutional neural network.

Despite extensive research in zero-watermarking, existing approaches still face several limitations. These issues can be summarized as follows:

1. Grayscale vs. Color Image Protection: Most traditional zero-watermarking techniques were designed for grayscale images, while color image protection is increasingly more relevant in real-world applications.
2. Weak Resistance to Combined Attacks: Many traditional zero-watermarking methods demonstrate weak resistance to combined signal-processing and geometric attacks, compromising the watermark’s robustness.
3. Instability in Moment-based Methods: Moment-based zero-watermarking approaches often rely on approximation methods, which can lead to instability, inaccuracy, and inefficiency, ultimately resulting in degraded feature extraction performance.
4. Weakness in Frequency Domain Feature Extraction: Feature extraction within the frequency domain is often ineffective against geometrical distortions and suffers from high time complexity, making it less practical for real-time applications.

To overcome the challenges outlined above, this paper presents a robust zero-watermarking approach that enhances resilience against various attacks while ensuring security and imperceptibility. Unlike traditional watermarking methods, zero watermarking protects copyright without embedding visible or hidden markers into the content. The proposed method integrates LBP with deep features from the CONV5-4 layer of VGG19, capturing both local texture details and high-level semantic information. This hybrid approach significantly enhances resistance to geometric distortions, signal processing attacks, and their combinations, including noise, scaling, and compression. Security is enhanced through frequency domain transformations and chaotic encryption based on the Lorenz system and the Logistic map. This framework strengthens multimedia security, providing a solid foundation for future research in digital information protection.

The main contributions of this paper are summarized as follows:

1. The proposed method integrates deep features from the CONV5-4 layer of VGG19 with LBP to enhance watermark resilience against various distortions and attacks. LBP extracts essential texture details, while VGG19 features capture high-level semantic representations to increase robustness.
2. The DWT decomposes the image into frequency sub-bands, while the DCT selects key coefficients for embedding, further enhancing robustness against signal processing and geometric attacks.
3. A secure encryption mechanism combines the Lorenz system and 2D Logistic Adjusted Chaotic Map (2D-LACM) to scramble the extracted features and the watermark data. The final zero watermark is generated through an XOR operation, ensuring ownership verification and strong resistance to compression, filtering, and transformation attacks.
4. The proposed approach achieves near-optimal results in terms of BER (0) and NCC (1), while maintaining high PSNR, thereby preserving image quality and watermark robustness.

The novelty of this work lies in the following aspects:

1. The unique fusion of VGG19 deep features and LBP in a zero-watermarking framework, which has not been explored in previous literature, provides complementary strengths for texture and semantic analysis.
2. The application of both DWT and DCT in a unified framework to address vulnerabilities in both spatial and frequency domains.
3. Applying a hybrid chaotic encryption system (Lorenz and 2D-LACM) to the watermark and the feature matrix introduces an extra security layer not typically present in existing watermarking methods.
4. Experimental results demonstrate that the proposed method consistently achieves near-perfect robustness and high imperceptibility under a wide range of attacks, significantly outperforming existing approaches in the literature.

The rest of the paper is structured as follows. “Proposed method” section outlines the fundamentals of the proposed method. “Results and analysis of experiments” section presents the results and analysis of experiments. Finally, “Conclusion” section concludes the paper.

## Proposed method

**Fig. 1:**
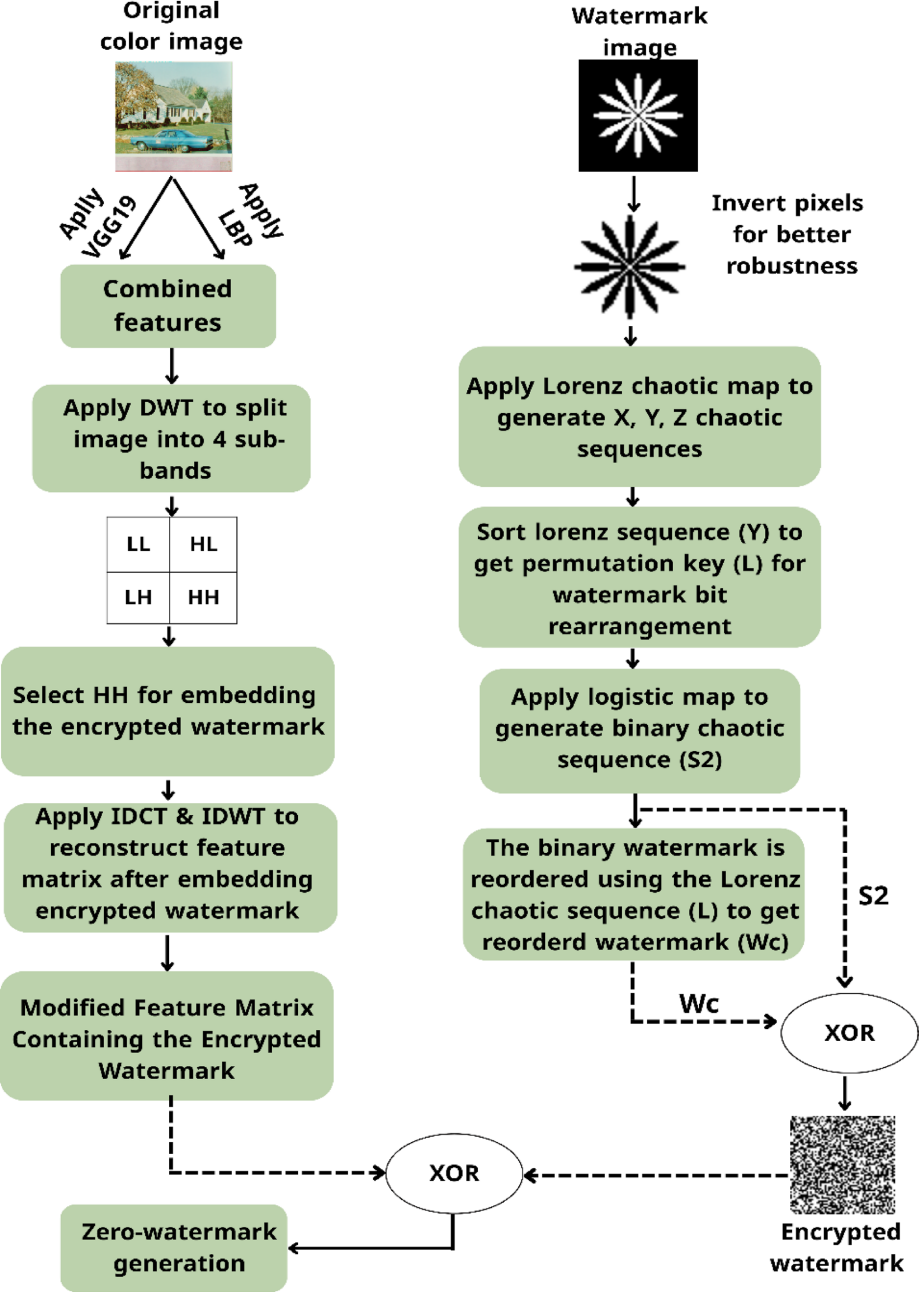
Process flow for zero-watermark generation.

**Fig. 2:**
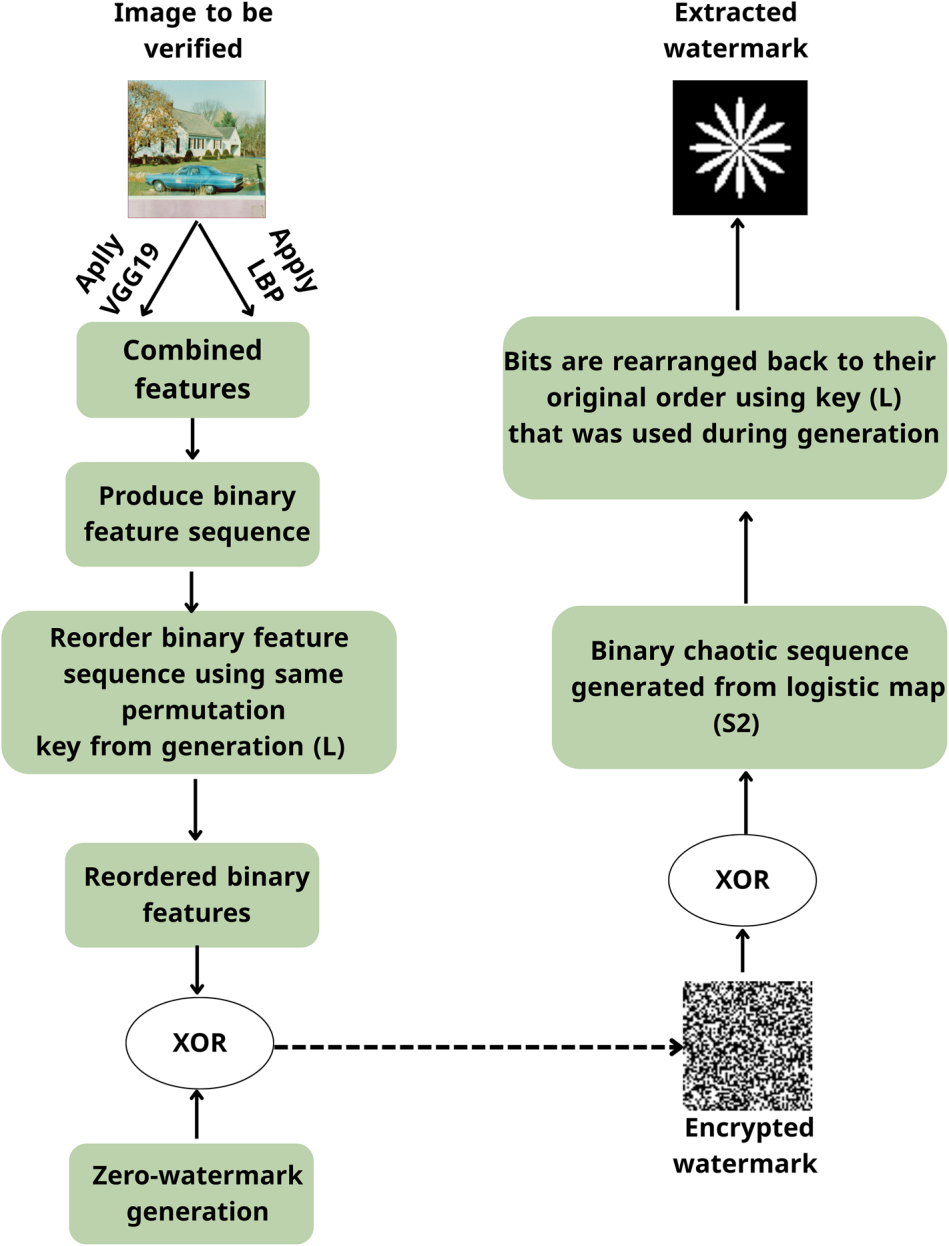
Process flow for zero-watermark verification.

The proposed scheme, Hybrid Deep-Chaotic Zero Watermarking Scheme, is designed to achieve robust and imperceptible zero watermarking by combining deep learning-based feature extraction with chaotic encryption. Figures 1 and 2 illustrate the overall workflow of the proposed method, which consists of two main phases: zero-watermark generation and zero-watermark verification.

The host and watermark images are input into the system in the generation phase. Features are extracted from the host image using a hybrid approach that combines Local Binary Patterns (LBP) and deep features obtained from the VGG19 model. These features are fused and binarized to form a robust binary feature matrix. Meanwhile, the watermark image is preprocessed by inverting its pixel values to improve robustness, then encrypted using a combination of Lorenz and Logistic chaotic maps. The resulting encrypted watermark is embedded into the high-frequency (HH) sub-band of the feature matrix, utilizing a combination of DWT and DCT. After embedding, the inverse transforms (IDCT and IDWT) are applied to reconstruct the modified feature matrix. Finally, a zero-watermark is generated by performing an XOR operation between the reconstructed matrix and the encrypted watermark.

In the verification phase, the attacked image undergoes the same feature extraction process using LBP and VGG19. The resulting binary feature matrix is reordered using the same chaotic permutation key employed during watermark generation. This reordered matrix is XOR-ed with the stored zero-watermark to recover the encrypted watermark. Decryption is then performed using the binary chaotic sequence to retrieve the original watermark. The extracted watermark is compared to the original watermark to assess the robustness and imperceptibility of the scheme under various common image processing attacks, including JPEG compression, rotation, scaling, filtering, cropping, translation, and noise addition. The detailed implementation steps and corresponding mathematical formulations are presented in the following subsections.

### Reading the host image and the watermark image

The suggested zero-watermarking technique begins by reading a color image (I) with size M × N, where M = N = 512. A binary watermark image (WI) is also used, with a size of m × n, where m = n = 64. These images serve as inputs for feature extraction and watermark embedding.

### Feature extraction

Feature extraction is crucial in watermarking, as it ensures resilience against various attacks. The proposed method extracts features from the host image using two complementary techniques: LBP for texture representation and VGG19 deep learning features for high-level semantic information.

- LBP

LBP is a widely used texture descriptor that encodes the local structure of an image by comparing pixel intensity values with those of their neighbors^35^. The image is divided into regions, and LBP values are extracted and combined into a global representation. The LBP value for a given pixel is computed as follows^36^:

1 $$S\left( {p,c} \right) = \left\{ {\begin{array}{*{20}l} {1,} \hfill & {if\;g\left( p \right) \ge g\left( c \right)} \hfill \\ {0,} \hfill & {otherwise} \hfill \\ \end{array} } \right.$$

**Fig. 3:**
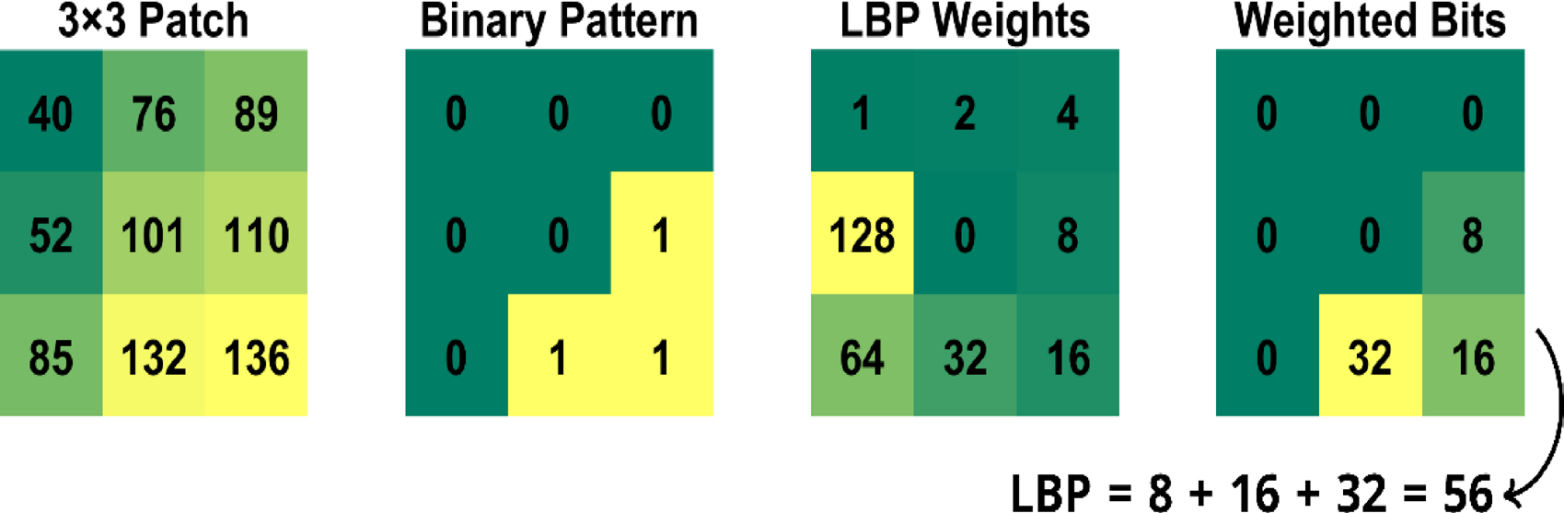
Illustration of LBP calculation on a 3 × 3 patch extracted from the image.

Here, g(p) and g(c) denote the pixel intensities of the neighboring and center pixels, respectively. This process enables texture analysis while ensuring robustness against variations in illumination. To illustrate the LBP computation process, a sample 3 × 3 patch is extracted from the “House” image, which is 512 × 512 in size, centered at pixel (50, 50), as shown in Fig. 3. The center pixel has a value of 101. A binary pattern is generated by comparing each neighboring pixel with the center pixel: if the neighbor is greater than or equal to 101, a binary 1 is assigned; otherwise, a binary 0 is assigned.

The resulting binary matrix is then multiplied element-wise by a predefined weight matrix, following the clockwise order of the neighbors starting from the top-left. Finally, the sum of the weighted binary values yields the LBP value, which is 56 in this case.

**Fig. 4:**
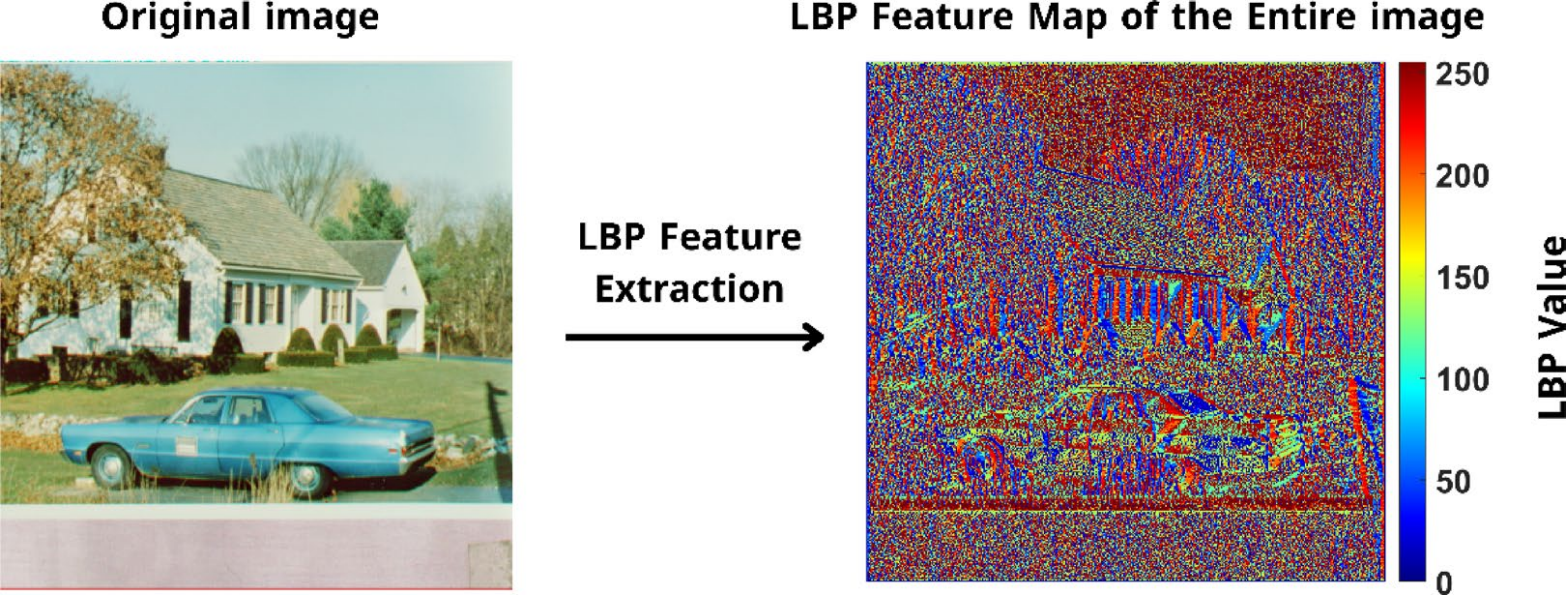
LBP feature map of the “house” image, showing pixel-wise texture encoding.

After computing the LBP value for a single pixel in a 3 × 3 patch, the same operation was systematically applied across the entire “House” image using a sliding 3 × 3 window as shown in Fig. 4. For each non-border pixel, the LBP value is calculated based on its local neighborhood using the same procedure previously illustrated in the 3 × 3 patch example. The process is repeated across the image, producing a matrix of LBP values that encodes local texture and edge information at each position, forming the LBP feature map.

Figure 4 visualizes the full LBP feature map using a color map, where each pixel’s color reflects its computed LBP value. Blue regions correspond to low LBP values, indicating smoother or low-contrast areas with less texture variation. Red and yellow regions indicate high LBP values, typically found in textured regions, object boundaries, and fine details.

This complete LBP map effectively captures the spatial distribution of textural features. It is later fused with high-level semantic features extracted from the VGG19 network to enhance the robustness and discrimination capability of the proposed hybrid zero-watermarking system.

- VGG19 Deep Features (Using the CONV5-4 Layer):

The proposed scheme incorporates deep semantic features using the pre-trained VGG19 convolutional neural network and local texture descriptors.

VGG19, developed by Simonyan and Zisserman in 2015^37^, is a deep CNN comprising 16 convolutional layers and 3 fully connected layers, totaling 19 layers. It is widely adopted for hierarchical feature extraction and image classification due to its uniform architecture and strong generalization capabilities^38^. The feature can be extracted by:

2 $${E}_{i}^{out}=\tau \left({H}_{i}*{E}_{i}^{in}+{d}_{i}\right)$$

Here, ${E}_{i}^{out}$ and ${E}_{i}^{in}$, are the input and output feature maps, H_i​_ is the convolutional filter, ${d}_{i}$, is the bias, and $\tau$, is the ReLU activation function. Pooling layers reduce computational complexity while preserving important details of the features. The final classification is performed using a softmax layer:

3 $${F}_{j}=\frac{{e}^{{y}_{j}}}{\sum_{c=1}^{C}{e}^{{y}_{c}}}$$

where ${F}_{j}$ represents the probability of a class $\text{j}$, and $\text{C}$ is the number of classes.

**Fig. 5:**
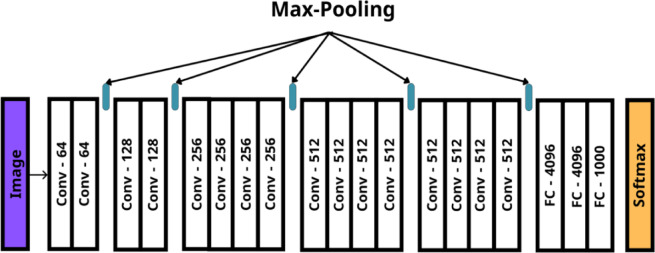
VGG19 architecture used for feature map extraction.

In our zero-watermarking approach, we utilize the VGG19 network architecture, as illustrated in Figure 5. Feature extraction is performed by passing the input image through the network to the conv5_4 layer, the last convolutional layer in the fifth block. The output of this layer is selected as the feature map because it provides high-level semantic representations that are more robust to common image transformations and distortions.

Figure 5 shows the internal structure of the VGG19 network used in this work. The model comprises five convolutional blocks, each followed by a max-pooling operation. As the image progresses through the network, the depth of the feature maps increases, allowing the network to learn increasingly abstract visual representations. In the proposed scheme, feature maps are extracted from the conv5_4 layer, highlighted in the final block, as it retains rich semantic information needed for robust zero-watermark generation.

Feature Fusion

The extracted LBP and VGG19 features are fused into a single feature vector to construct a robust and discriminative representation. The LBP captures fine-grained local texture information, while VGG19 provides high-level semantic context. Combining these complementary features enables the system to leverage the image’s regional and global characteristics. This hybrid representation significantly enhances the system’s resilience against various image distortions and attacks, including noise, filtering, compression, and geometric transformations.

### Watermark embedding

The Lorenz system and the Logistic map generate chaotic sequences that provide strong encryption for the watermark, ensuring both robustness and security. The watermark is incorporated into the high-frequency components of the image using the DCT and the DWT, followed by encryption using the Exclusive-OR (XOR) operation. This ensures that the watermark is safely and invisibly embedded while maintaining the quality of the original image. Each step is explained in detail below.

1. Chaotic Encryption Using Lorenz System and Logistic Map:

To improve security, the watermark undergoes encryption using two chaotic maps:

Lorenz system: a chaotic system used for scrambling the watermark features.

Logistic Map: a chaotic system used to generate binary sequences.

*Step 1* Generating a Chaotic Sequence with the Lorenz System

The Lorenz system, introduced in 1963, is a chaotic system represented by the following differential equations^39^:

4 $$\left\{\begin{array}{c}\frac{dx}{dt}=\sigma \left(y-x\right)\\ \frac{dy}{dt}=x\left(\rho -z\right)-y\\ \frac{dz}{dt}=xy-\beta z\end{array}\right.$$

For parameters $\sigma$ = 10, ρ = 28, and β = $\frac{8}{3}$, the system exhibits chaotic behavior, making it ideal for encryption due to its sensitivity to initial conditions and unpredictability. The chaotic properties enable secure scrambling of features and watermark data. The initial conditions are selected as follows:

$$x\left(0\right)=0.8633, y\left(0\right)=0.9234, z\left(0\right)=0.1$$

The values of $\text{x}$, $\text{y}$, and $\text{z}$ are then calculated over time using the previous formulae, resulting in a chaotic sequence that is utilized to scramble the watermark:

5 $$\left\{\begin{array}{c}{x}_{i+1}={x}_{i}+\sigma \cdot \left({y}_{i}-{x}_{i}\right)\cdot dt\\ {y}_{i+1}={y}_{i}+\left({x}_{i}\cdot \left(\rho -{z}_{i}\right)-{y}_{i}\right)\cdot dt\\ {z}_{i+1}={z}_{i}+\left({x}_{i}\cdot {y}_{i}-\beta \cdot {z}_{i}\right)\cdot dt\end{array}\right.$$

The initial values $x\left(1\right), y\left(1\right),$ and $z\left(1\right)$ are saved as SK1 (chaotic key).

*Step 2* Generating a Chaotic Sequence with the Logistic Map

The 1D logistic map is another chaotic function that produces pseudo-random sequences^40^.

This map is used to construct another chaotic sequence, further scrambling the watermark. The logistic map equation is presented below:

6 $${x}_{n+1}=\mu \cdot {x}_{n}\cdot \left(1-{x}_{n}\right)$$

Here, ${x}_{n}$ is the current value within the range [0, 1], while $\mu$ is the control parameter ranges from 0 to 4. The logistic map exhibits chaotic behavior when $\mu$ falls within the range (3.569945972, …, 4]. Therefore, it is typically set to **3.999** to ensure a highly chaotic sequence.

The generated sequence is then thresholded, resulting in a binary sequence suitable for encryption.

7 $${S}_{2}=\text{Logistic Sequence}\ge \text{mean(Logisti}\text{c Sequence)}$$

## Watermark embedding using DWT and DCT

*Step 1* Decomposition using DWT

The input image is decomposed using DWT into four frequency sub-bands: $LL$, $LH$, $HL$, and $HH$. The $HH$ A subband containing high-frequency components is selected for embedding the watermark. This band strikes a balance between imperceptibility and sensitivity to specific types of attacks.

*Step 2* Applying DCT

DCT is then applied to the HH sub-band. This transformation concentrates the image’s energy into a small number of significant coefficients, allowing the watermark to be embedded in a compact yet robust manner. By embedding the watermark in the frequency domain rather than directly in the spatial domain, it becomes more resistant to compression and filtering attacks. This combination of DWT and DCT ensures imperceptibility and robustness, making the embedding process suitable for zero watermarking applications.

*Step 3* Chaotic Encryption of the Watermark.

The watermark undergoes additional security measures before embedding:

1. Inversion and Flattening:
  The watermark is inverted to increase security. The inversion is mathematically represented as:
    8 $${W}_{b}={\text{reshape}}\left(\sim WI,[1,m\cdot n]\right)$$
    where $\sim WI,$ represents the bitwise inversion of the watermark.

2. Chaotic Reordering:
  The binary watermark is reordered utilizing the Lorenz chaotic sequence:
    9 $${W}_{c}={W}_{b}\left(L\right)$$

3. XOR-based Encryption:
  The reordered watermark is encrypted using XOR with the Logistic chaotic sequence:
    10 $${W}_{\text{en}}={\text{xor}}\left({W}_{c},S2\right)$$

The encrypted watermark is reshaped back to a 2D matrix:

11 $$W{I}_{\text{encrypted}}={\text{reshape}}\left({W}_{\text{en}},[m,n]\right)$$

*Step 4* Embedding the Encrypted Watermark.

The encrypted watermark is incorporated within the DCT coefficients of the HH sub-band. This process modifies the DCT coefficients to embed the watermark securely:

12 $$H{H}_{\text{dct}}\left(x,y\right)=H{H}_{\text{dct}}\left(x,y\right)+\alpha \cdot W{I}_{\text{encrypted}}\left(x,y\right)$$

Here, $\alpha$ is the embedding strength factor.

*Step 5* Inverse Transformations (IDCT and IDWT)

After embedding the encrypted watermark into the DCT coefficients of the HH subband, the Inverse Discrete Cosine Transform (IDCT) is applied to transform the frequency-domain data back to the spatial domain. Subsequently, the Inverse Discrete Wavelet Transform (IDWT) is used to reconstruct the full watermarked image by combining the modified HH subband with the original LL, LH, and HL subbands. These inverse transformations ensure that the watermarked image retains high visual quality while preserving the embedded watermark.

Final Watermark Embedding Using XOR:

To further secure the watermark, an XOR operation is applied between the watermarked image and the encrypted watermark:

13 $${W}_{\text{zero}}={\text{xor}}\left(\text{Watermarked Image},{\text{WI}}_{\text{encrypted}}\right)$$

This ensures that the watermark remains imperceptible while resisting various attacks.

### Watermark extraction

The watermark extraction process aims to accurately recover the embedded watermark from an attacked image while preserving its integrity and authenticity. This is achieved through XOR-based decryption and inverse chaotic scrambling using the Lorenz system sequence.

The extraction process follows these steps:

XOR Decryption: The encoded watermark is decrypted by executing an XOR operation with the binary chaotic sequence $S2$:
  14 $$WI\left(x,y\right)={W}_{e}\left(x,y\right)\oplus S2\left(x,y\right)$$
  where $WI(x,y)$ is the extracted watermark, ${W}_{e}(x,y)$ is the encrypted watermark from the attacked image, and $S2(x,y)$, is the chaotic sequence.
Restoring the Original Order: To reconstruct the original watermark, inverse chaotic scrambling is applied using the Lorenz system sequence $\text{L}$. This reverses the initial scrambling performed during the embedding phase.
(3) Reshaping the Watermark: The binary watermark is reshaped into a matrix of size $m\times n$, to match the original watermark dimensions.

This ensures accurate recovery of the watermark while preserving its integrity against various attacks.

The steps of the proposed method are summarized as follows:
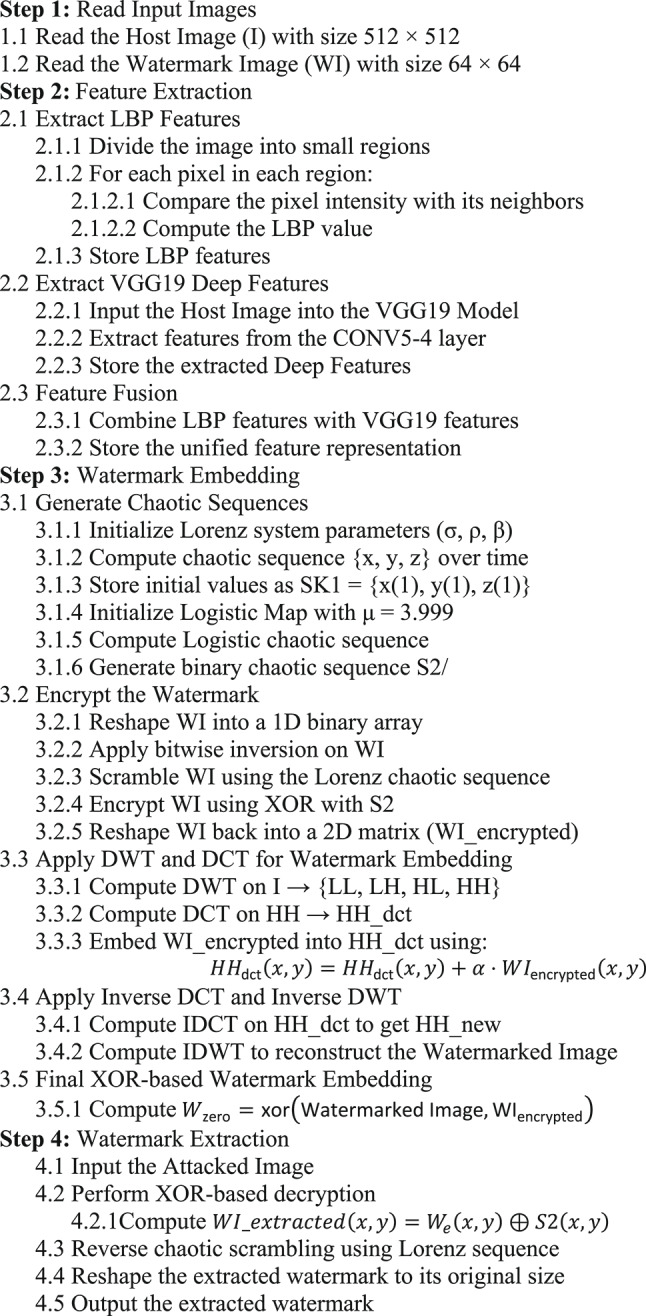


This methodology ensures robust, imperceptible, and secure zero-watermarking while achieving high resilience against attacks.

## Results and analysis of experiments

Generally, effective zero-watermarking necessitates both durability and invisibility. Zero-watermarking is exceptionally imperceptible by design, and durability is a critical requirement. In this section, we will conduct a series of tests to evaluate the efficiency of the zero-watermarking method proposed in this paper.

**Fig. 6:**
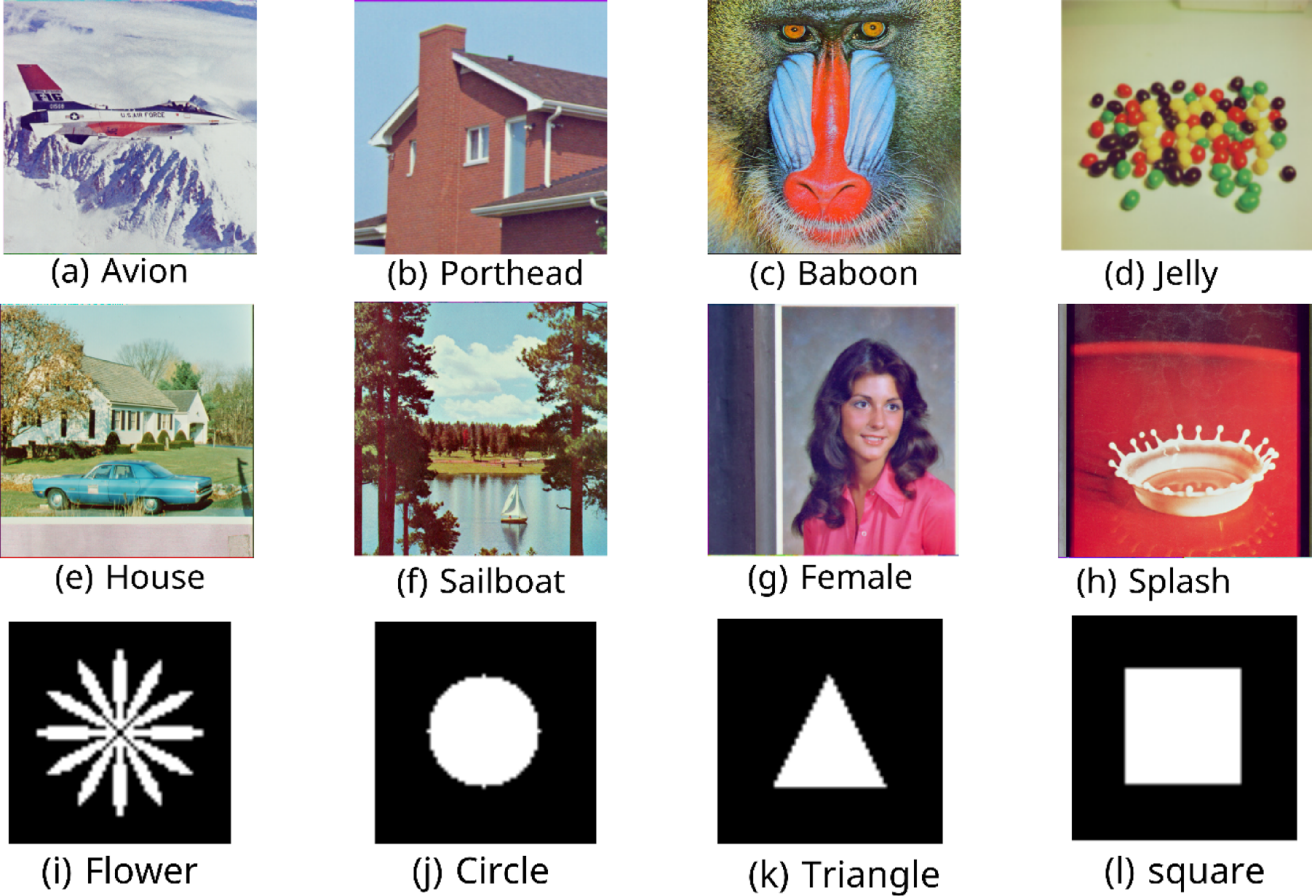
eight color images (**a**–**h**) and four binary images (**i**–**l**).

Experimental dataset: We selected eight 512 × 512-pixel color images from the USC-SIPI^41^ and the Computer Vision Group (CVG)^42^ datasets as the host images, as shown in Fig. 6a–h, and four binary images with 64 × 64-pixel watermarks, as shown in Fig. 6i–l.

*Parameter Setting* This paper’s suggested zero-watermarking approach parameters are as follows: the host image size is M = N = 512, and the watermark image size is m = n = 64. The Lorenz system’s initial state values and parameters are defined as ${x}_{0}=0.8633$, ${y}_{0}=0.9234$, ${z}_{0}=0.1$, $\sigma =10$, $\rho =28$, $\beta =\frac{8}{3}$ and the integration step $\Delta t=0.01$. The Logistic map control parameter is set as $\mu =3.999$. The watermark embedding strength factor is $\alpha =0.05$.

*Evaluation metrics* The proposed zero-watermarking approach utilizes the PSNR to assess the visual quality of the distorted color images compared to the originals. The PSNR is computed as follows:

15 $$\text{PSNR}=10{\text{log}}_{10}\left(\frac{{255}^{2}\times M\times N}{\sum_{h=1}^{3}\sum_{x=1}^{M}\sum_{y=1}^{N}{\left[{I}_{h}\left(x,y\right)-I{I}_{h}\left(x,y\right)\right]}^{2}}\right)$$

where ${I}_{h}\left(x,y\right)$ and ${II}_{h}\left(x,y\right)$, represent the original and distorted images of size $M \times N$, respectively, and $h\in \left\{R,G,B\right\},$ refers to the color channels.

The watermarking system’s resilience is evaluated using the BER and NCC. These measures calculate the similarity and error between the original and retrieved watermarks after attacks. The definitions are as follows:

16 $$BER = \frac{1}{m \times n}\mathop \sum \limits_{i = 1}^{m} \mathop \sum \limits_{j = 1}^{n} \left[ {WI\left( {i,j} \right) \oplus WI^{\prime } \left( {i,j} \right)} \right]$$

17 $$NCC = \frac{{\mathop \sum \nolimits_{i = 1}^{m} \mathop \sum \nolimits_{j = 1}^{n} \left[ {WI\left( {i,j} \right) \oplus WI^{\prime } \left( {i,j} \right)} \right]}}{{\sqrt {\mathop \sum \nolimits_{i = 1}^{m} \mathop \sum \nolimits_{j = 1}^{n} \left[ {WI\left( {i,j} \right)} \right]^{2} } \cdot \sqrt {\mathop \sum \nolimits_{i = 1}^{m} \mathop \sum \nolimits_{j = 1}^{n} \left[ {WI^{\prime } \left( {i,j} \right)} \right]^{2} } }}$$

where $WI\left(i,j\right)$ and $WI^{\prime } \left( {{\text{i}},{\text{j}}} \right)$ represent the original and extracted watermark images of size $m\times n$, respectively, and $\oplus$ denotes the XOR operation. A lower BER value increases the system’s robustness. Conversely, a higher NCC value indicates more remarkable similarity and improved robustness.

### Anti‑attack performance analysis

The suggested zero-watermark approach is tested against conventional attacks to ensure its robustness. Color images in this subsection are subjected to various attacks, including rotation, scaling, brightness adjustments, filtering process, noise, and JPEG compression. The extracted and original watermarks are compared to calculate the BER and NCC values. Table 1 provides thorough explanations of traditional signal-processing and geometrical attacks.

**Table 1:** Attack types with varying parameters.

Types of attacks	Parametric description of attacks
Filtering	Average filtering using window sizes of 3 × 3, 5 × 5, and 7 × 7
	Gaussian filtering using window sizes of 3 × 3, 5 × 5, and 9 × 9
	Median filtering using window sizes of 3 × 3, 5 × 5, and 7 × 7
	Wiener filtering using window sizes of 3 × 3, 5 × 5, and 7 × 7
Geometric transform	Rotation (1°, 3°, 5°, 10°, 50°) using nearest, bilinear, and bicubic interpolation
	Scaling (0.25, 0.5, 2.0, 4.0) using nearest, bilinear, and bicubic interpolation
JPEG compression	quality factor = 5, 10, 20, 30, 40, 50, 60, 70, 80, 90
Brightness adjustment	Histogram equalization
Noise	Gaussian noise with parameters 0.1, 0.2, 0.3, and 0.5
	Salt and pepper noise with parameters 0.1, 0.2, 0.3, and 0.5
Translation attack	factor = 10, 20, 40, 60
Conventional combined attacks	Median filter (5 × 5) + Gaussian noise (0.3)
	Median filter (5 × 5) + Salt & pepper noise (0.3)
	Wiener filter (5 × 5) + Salt & pepper noise (0.3)
	Median filter (5 × 5) + JPEG Compression (90)
	Gaussian noise (0.3) + JPEG Compression (90)
	Salt & pepper noise (0.3) + JPEG Compression (90)
	Rotation (2°) + JPEG Compression (10)
	Scaling (0.5) + JPEG Compression (90)
Center cropping attack	32 × 32, 64 × 64, 128 × 128
Top left cropping attack	32 × 32, 64 × 64, 128 × 128

This section evaluates the technique’s robustness against popular image processing and geometrical attacks. The tests performed might be split into two primary categories.

- We began with a 512 × 512 color image called “House,” as shown in Fig. 6. Tables 2, 3, 4, 5, 6, 7, 8, and 9 show the PSNR, BER, and NCC values for each suggested attack and the recovered watermark images. The watermark was created using a binary image named ‘Flower.’ These tables demonstrate that the proposed technique produces more similar watermarks than the original. The obtained BER and NCC values are near optimal.
- Experiments were conducted on seven standard color images, as shown in Fig. 6. Tables 10, 11, 12, 13, 14, and 15 present the values of PSNR and NCC for the proposed methods in response to each attack.

**Table 2:**
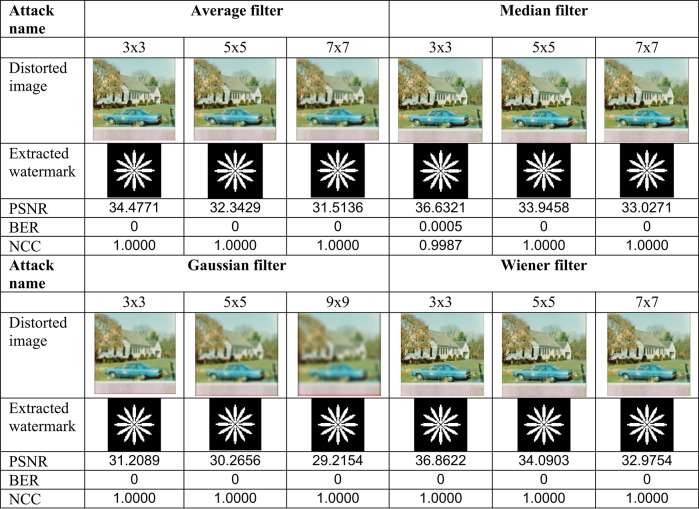
Evaluation of resistance to filtering attacks.

**Table 3:**
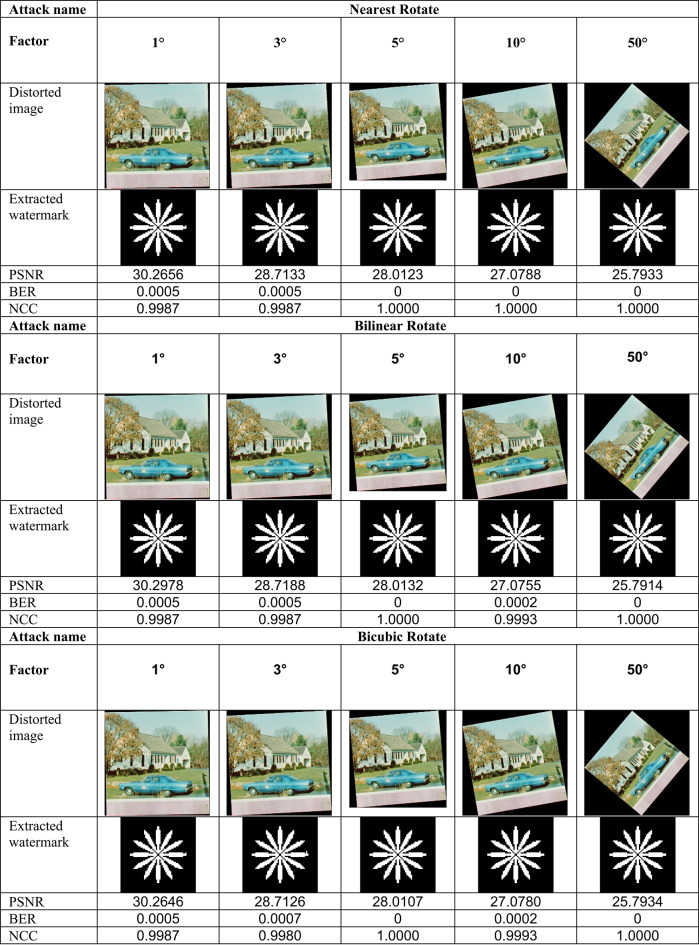
Evaluation of resistance to rotation attacks

**Table 4:**
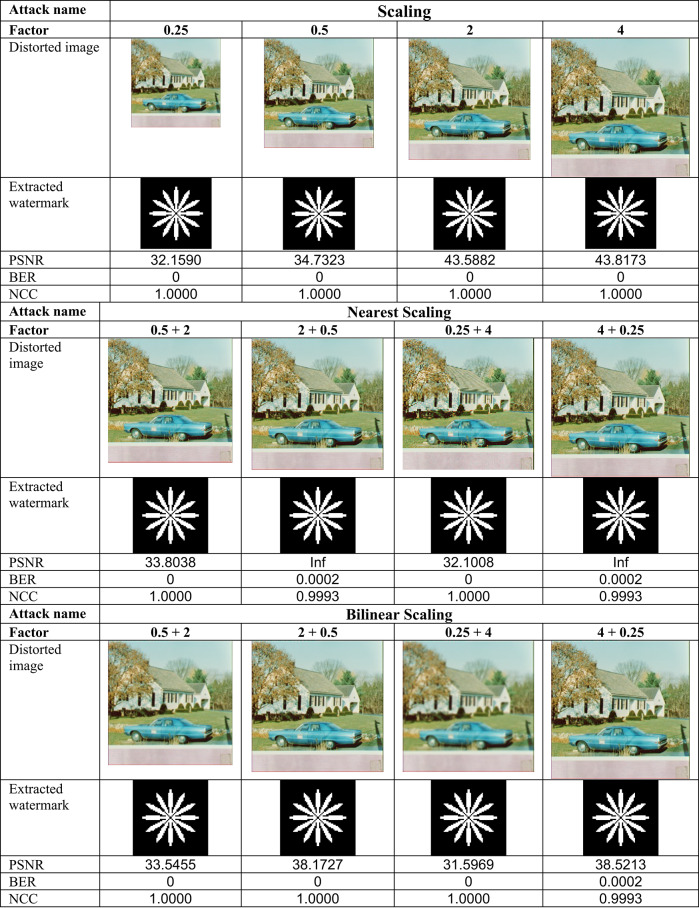

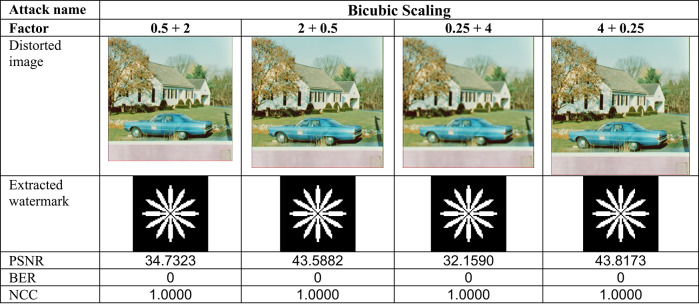
Evaluation of resistance to scaling attacks

**Table 5:**
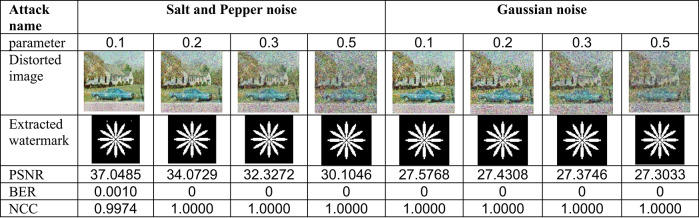
Evaluation of resistance to noise attacks

**Table 6:**
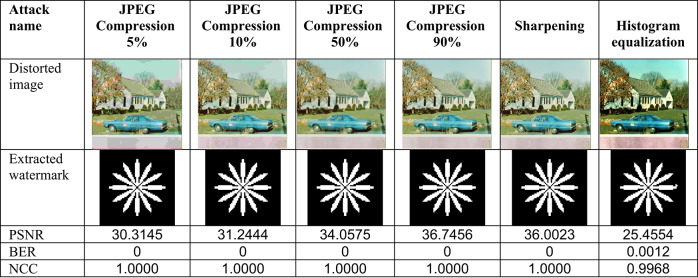
Evaluation of resistance to JPEG compression and brightness adjustment attacks

**Table 7:**
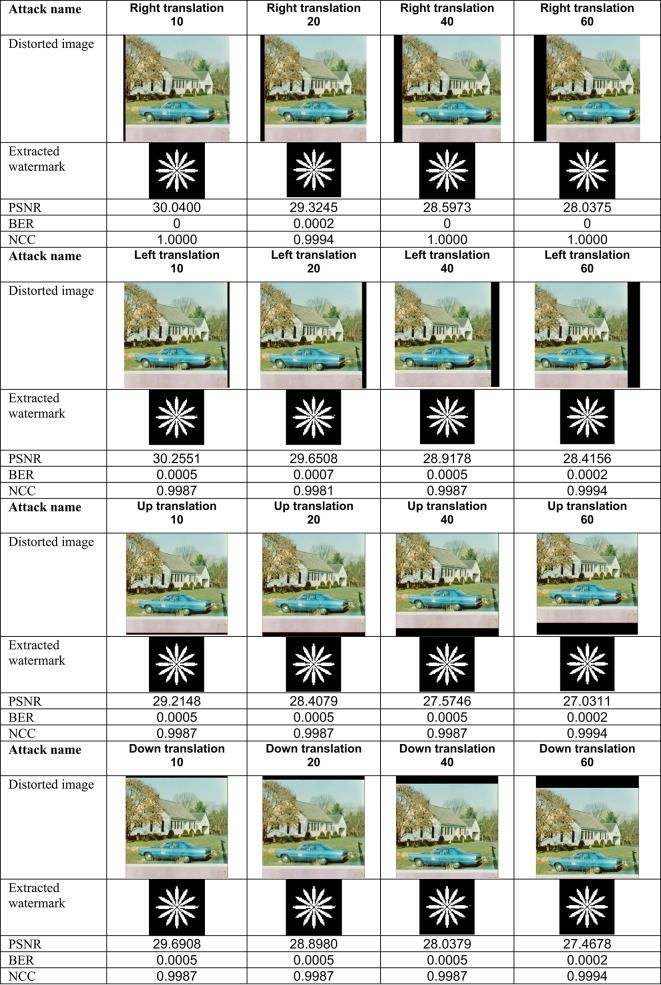
Evaluation of resistance to translation attacks

**Table 8:**
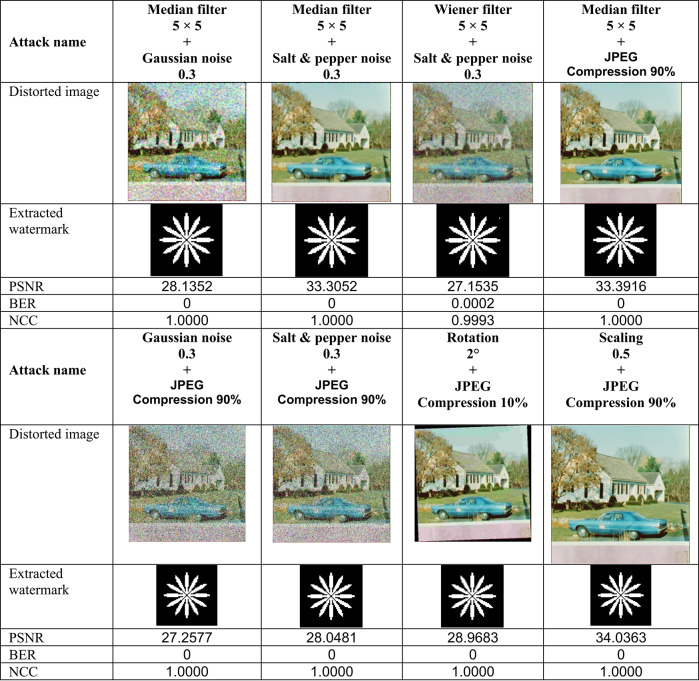
Evaluation of resistance to conventional combined attacks

**Table 9:**
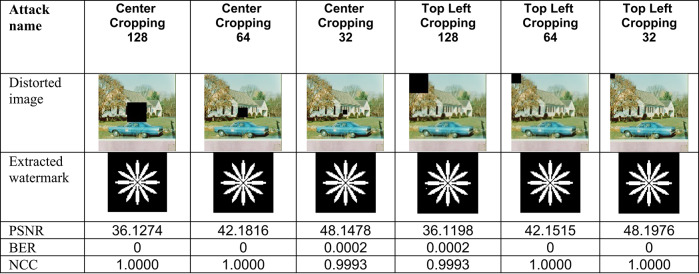
Evaluation of resistance to image cropping attacks

**Table 10:** Robustness to filtering attacks.

Attack name	Average filter				Median filter			
Factor	3 × 3		5 × 5	7 × 7	3 × 3		5 × 5	7 × 7
Avion	PSNR	36.9519	34.3550	33.1878	PSNR	40.0758	37.1999	35.9914
	NCC	1.0000	1.0000	1.0000	NCC	1.0000	1.0000	1.0000
	Gaussian Filter				Wiener Filter			
	3 × 3		5 × 5	9 × 9	3 × 3		5 × 5	7 × 7
	PSNR	42.5468	42.5266	42.5266	PSNR	40.6999	37.5362	35.8220
	NCC	1.0000	1.0000	1.0000	NCC	1.0000	1.0000	1.0000
Jelly	Average Filter				Median Filter			
	3 × 3		5 × 5	7 × 7	3 × 3		5 × 5	7 × 7
	PSNR	39.5067	35.7466	34.1959	PSNR	42.9774	38.1896	36.4463
	NCC	0.9997	0.9995	0.9997	NCC	1.0000	1.0000	1.0000
	Gaussian Filter				Wiener Filter			
	3 × 3		5 × 5	9 × 9	3 × 3		5 × 5	7 × 7
	PSNR	42.1588	42.1289	42.1288	PSNR	44.2677	39.5608	37.4058
	NCC	1.0000	1.0000	1.0000	NCC	0.9996	0.9996	0.9996
Baboon	Average Filter				Median Filter			
	3 × 3		5 × 5	7 × 7	3 × 3		5 × 5	7 × 7
	PSNR	30.5574	29.8525	29.5626	PSNR	31.3107	30.1915	29.8868
	NCC	0.9991	1.0000	1.0000	NCC	0.9986	1.0000	1.0000
	Gaussian Filter				Wiener Filter			
	3 × 3		5 × 5	9 × 9	3 × 3		5 × 5	7 × 7
	PSNR	34.5939	34.5789	34.5789	PSNR	31.5090	30.4939	30.1315
	NCC	1.0000	1.0000	1.0000	NCC	0.9991	0.9991	1.0000
Sailboat	Average Filter				Median Filter			
	3 × 3		5 × 5	7 × 7	3 × 3		5 × 5	7 × 7
	PSNR	33.4433	32.2868	31.6239	PSNR	34.1446	32.9641	32.3433
	NCC	1.0000	1.0000	1.0000	NCC	1.0000	1.0000	1.0000
	Gaussian Filter				Wiener Filter			
	3 × 3		5 × 5	9 × 9	3 × 3		5 × 5	7 × 7
	PSNR	38.8819	38.8646	38.8646	PSNR	35.2778	34.0086	33.1500
	NCC	1.0000	1.0000	1.0000	NCC	1.0000	1.0000	1.0000
Female	Average Filter				Median Filter			
	3 × 3		5 × 5	7 × 7	3 × 3		5 × 5	7 × 7
	PSNR	42.4316	37.8891	35.7977	PSNR	46.7506	41.7213	39.5946
	NCC	1.0000	1.0000	1.0000	NCC	1.0000	1.0000	1.0000
	Gaussian Filter				Wiener Filter			
	3 × 3		5 × 5	9 × 9	3 × 3		5 × 5	7 × 7
	PSNR	35.4898	33.2502	31.4611	PSNR	47.7277	42.4023	39.7629
	NCC	1.0000	1.0000	1.0000	NCC	1.0000	1.0000	1.0000
Splash	Average Filter				Median Filter			
	3 × 3		5 × 5	7 × 7	3 × 3		5 × 5	7 × 7
	PSNR	39.1545	36.8348	35.6285	PSNR	41.5037	39.5480	38.3779
	NCC	1.0000	1.0000	1.0000	NCC	1.0000	1.0000	1.0000
	Gaussian Filter				Wiener Filter			
	3 × 3		5 × 5	9 × 9	3 × 3		5 × 5	7 × 7
	PSNR	35.1670	33.4577	32.1496	PSNR	42.3717	40.3789	39.1574
	NCC	1.0000	1.0000	1.0000	NCC	1.0000	1.0000	1.0000
Porthead	Average Filter				Median Filter			
	3 × 3		5 × 5	7 × 7	3 × 3		5 × 5	7 × 7
	PSNR	41.4144	36.7763	34.7859	PSNR	46.5120	40.1981	37.7902
	NCC	1.0000	1.0000	1.0000	NCC	1.0000	1.0000	1.0000
	Gaussian Filter				Wiener Filter			
	3 × 3		5 × 5	9 × 9	3 × 3		5 × 5	7 × 7
	PSNR	45.6489	45.6319	45.6319	PSNR	46.0645	40.5581	38.1893
	NCC	1.0000	1.0000	1.0000	NCC	1.0000	1.0000	1.0000

**Table 11:** Robustness to Rotation-based attacks.

Attack name	Bilinear interpolation rotation					Bicubic interpolation rotation				
Factor	1°		3°	5°	10°	1°		3°	5°	10°
Avion	PSNR NCC	31.7638 1.0000	29.7367 1.0000	28.8379 1.0000	27.5618 1.0000	PSNR NCC	31.7314 1.0000	29.7341 1.0000	28.8410 1.0000	27.5677 1.0000
Jelly	PSNR NCC	31.2574 0.9998	28.8842 0.9995	27.9329 0.9995	26.8331 1.0000	PSNR NCC	31.2153 0.9998	28.8852 0.9995	27.9381 0.9995	26.8317 1.0000
Baboon	PSNR NCC	28.6955 0.9987	27.8239 0.9987	27.3698 0.9987	26.6522 1.0000	PSNR NCC	28.6481 0.9987	27.8035 0.9987	27.3577 0.9987	26.6451 1.0000
Sailboat	PSNR NCC	30.1571 1.0000	28.6563 1.0000	27.9703 1.0000	27.0277 1.0000	PSNR NCC	30.0975 1.0000	28.6342 1.0000	27.9585 1.0000	27.0226 1.0000
Female	PSNR NCC	33.0800 1.0000	29.8908 1.0000	28.6550 1.0000	27.2056 1.0000	PSNR NCC	33.0587 1.0000	29.8844 1.0000	28.6514 1.0000	27.2039 1.0000
Splash	PSNR NCC	32.6833 1.0000	29.9170 1.0000	28.8364 1.0000	27.4139 1.0000	PSNR NCC	32.6279 1.0000	29.8966 1.0000	28.8234 1.0000	27.4082 1.0000
Porthead	PSNR NCC	32.4308 1.0000	30.1688 1.0000	29.2069 1.0000	27.7619 1.0000	PSNR NCC	32.3773 1.0000	30.1481 1.0000	29.1942 1.0000	27.7557 1.0000

**Table 12:** Robustness to scaling attacks.

Attack name	Nearest interpolation				
Factor	0.5 + 2		2 + 0.5	0.25 + 4	4 + 0.25
Avion	PSNR NCC	36.0454 1.0000	Inf 1.0000	34.2342 1.0000	Inf 1.0000
Jelly	PSNR NCC	36.9019 0.9990	Inf 0.9996	34.7644 1.0000	Inf 0.9996
Baboon	PSNR NCC	30.7370 0.9995	Inf 0.9973	29.5453 1.0000	Inf 0.9973
Sailboat	PSNR NCC	32.7591 1.0000	Inf 1.0000	31.5071 1.0000	Inf 1.0000
Female	PSNR NCC	39.8449 1.0000	Inf 1.0000	36.5545 1.0000	Inf 1.0000
Splash	PSNR NCC	38.3154 1.0000	Inf 1.0000	36.4175 1.0000	Inf 1.0000
Porthead	PSNR NCC	39.0284 1.0000	Inf 1.0000	35.7365 1.0000	Inf 1.0000
Bilinear interpolation					
Avion	PSNR NCC	35.9792 1.0000	41.0237 1.0000	33.5279 1.0000	41.3388 1.0000
Jelly	PSNR NCC	38.3378 0.9995	45.5895 0.9995	35.0016 1.0000	45.9576 0.9995
Baboon	PSNR NCC	30.4251 0.9991	32.3272 0.9991	29.7106 1.0000	32.5467 0.9991
Sailboat	PSNR NCC	33.1864 1.0000	36.2117 1.0000	31.7778 1.0000	36.4816 1.0000
Female	PSNR NCC	41.9500 1.0000	50.7906 1.0000	36.3490 1.0000	51.1281 1.0000
Splash	PSNR NCC	38.6732 1.0000	43.0935 1.0000	35.8353 1.0000	43.3467 1.0000
Porthead	PSNR NCC	40.7395 1.0000	49.1285 1.0000	35.8359 1.0000	49.3445 1.0000
Bicubic interpolation					
Avion	PSNR NCC	37.4623 1.0000	46.2194 1.0000	34.2027 1.0000	46.5612 1.0000
Jelly	PSNR NCC	41.3696 0.9996	54.1246 0.9995	36.0855 0.9991	55.3283 0.9996
Baboon	PSNR NCC	30.7058 0.9991	35.7239 0.9986	29.8809 1.0000	35.9024 0.9986
Sailboat	PSNR NCC	33.7986 1.0000	40.1902 1.0000	32.1883 1.0000	40.3911 1.0000
Female	PSNR NCC	47.7747 1.0000	58.8018 1.0000	37.8796 1.0000	60.7623 1.0000
Splash	PSNR NCC	39.7790 1.0000	47.6179 1.0000	36.6511 1.0000	48.0472 1.0000
Porthead	PSNR NCC	46.2508 1.0000	57.3491 1.0000	37.0242 1.0000	59.1332 1.0000

**Table 13:** Robustness to noise attacks.

Attack name	Gaussian noise					Salt and Pepper noise				
Parameter	0.1		0.2	0.3	0.5	0.1		0.2	0.3	0.5
Avion	PSNR NCC	27.5622 1.0000	27.4190 0.9995	27.3569 1.0000	27.2931 1.0000	PSNR NCC	37.1221 1.0000	34.0713 1.0000	32.3246 0.9986	30.9049 1.0000
Jelly	PSNR NCC	27.5719 0.9991	27.4467 1.0000	27.4748 1.0000	27.3145 1.0000	PSNR NCC	37.1286 0.9992	34.1039 1.0000	32.3538 1.0000	30.1987 1.0000
Baboon	PSNR NCC	27.5714 0.9977	27.4161 1.0000	27.3609 1.0000	27.2996 1.0000	PSNR NCC	37.0975 0.9986	34.0943 1.0000	32.3122 0.9995	30.0949 1.0000
Sailboat	PSNR NCC	27.6036 1.0000	27.4707 0.9994	27.4007 1.0000	27.3441 1.0000	PSNR NCC	37.1370 1.0000	34.1134 1.0000	32.3522 1.0000	30.1447 1.0000
Female	PSNR NCC	27.5754 1.0000	27.4273 1.0000	27.3568 1.0000	27.2968 1.0000	PSNR NCC	37.0678 0.9974	34.0991 0.9981	32.2992 1.0000	30.1175 1.0000
Splash	PSNR NCC	27.7001 1.0000	27.5581 1.0000	27.4918 1.0000	27.4171 1.0000	PSNR NCC	37.2528 1.0000	34.2030 1.0000	32.4556 1.0000	30.2135 1.0000
Porthead	PSNR NCC	27.5666 0.9974	27.4203 1.0000	27.3393 1.0000	27.2897 1.0000	PSNR NCC	37.0708 1.0000	34.0890 1.0000	32.3048 1.0000	30.1034 1.0000

**Table 14:** Robustness against JPEG compression attacks.

Attack name	JPEG compression					
Factor	5%		10%	20%	30%	40%
Avion	PSNR NCC	31.3763 1.0000	32.8542 1.0000	34.3613 1.0000	35.2268 1.0000	35.7641 1.0000
Jelly	PSNR NCC	30.2356 1.0000	33.0536 1.0000	35.2762 0.9995	36.5447 0.9991	37.3929 0.9990
Baboon	PSNR NCC	29.1392 1.0000	29.5760 1.0000	30.0987 1.0000	30.4482 1.0000	30.6521 0.9991
Sailboat	PSNR NCC	29.9892 1.0000	30.9633 1.0000	31.9154 1.0000	32.3637 1.0000	32.6343 1.0000
Female	PSNR NCC	30.9916 1.0000	33.3499 1.0000	36.0481 0.9994	37.6515 1.0000	38.4897 1.0000
Splash	PSNR NCC	30.9805 1.0000	33.0482 1.0000	35.0845 1.0000	35.9949 1.0000	36.5801 1.0000
Porthead	PSNR NCC	32.1182 1.0000	34.0507 1.0000	35.8001 1.0000	37.3113 1.0000	37.7567 1.0000

Attack name	JPEG compression					
Factor	50%		60%	70%	80%	90%
Avion	PSNR NCC	36.1267 1.0000	36.5733 1.0000	37.0820 1.0000	37.8384 1.0000	39.1233 1.0000
Jelly	PSNR NCC	37.8213 0.9997	38.3442 0.9992	38.9532 0.9996	39.8781 0.9991	41.4739 0.9996
Baboon	PSNR NCC	30.8591 0.9991	31.0708 0.9991	31.3743 0.9982	31.8324 0.9977	32.7037 0.9973
Sailboat	PSNR NCC	32.8419 1.0000	33.0350 1.0000	33.2845 1.0000	33.6402 1.0000	34.3908 1.0000
Female	PSNR NCC	39.1666 1.0000	40.1298 1.0000	40.9832 1.0000	42.0596 1.0000	43.8653 1.0000
Splash	PSNR NCC	36.9447 1.0000	37.4013 1.0000	37.9781 1.0000	38.7025 1.0000	39.8652 1.0000
Porthead	PSNR NCC	38.5223 1.0000	39.2416 1.0000	39.9404 1.0000	40.7650 1.0000	42.3187 1.0000

**Table 15:** Robustness against conventional combined attacks.

Attack name	Median filter (5 × 5) + Gaussian noise 0.3		Median filter (5 × 5) + Salt & pepper noise 0.3	Wiener filter (5 × 5) + Salt & pepper noise 0.3	Median filter (5 × 5) + JPEG Compression 90
Avion	PSNR NCC	28.1524 1.0000	36.0537 1.0000	26.1368 1.0000	36.4525 1.0000
Jelly	PSNR NCC	28.2193 1.0000	36.6889 1.0000	28.8964 1.0000	37.4938 1.0000
Baboon	PSNR NCC	28.0134 1.0000	30.0195 0.9991	28.3395 1.0000	30.0418 0.9991
Sailboat	PSNR NCC	28.1634 1.0000	32.5795 1.0000	28.4654 1.0000	32.6563 1.0000
Female	PSNR NCC	28.1695 1.0000	39.1808 0.9994	29.8782 1.0000	40.6152 1.0000
Splash	PSNR NCC	28.3226 1.0000	38.1297 1.0000	29.5345 1.0000	38.1674 1.0000
Porthead	PSNR NCC	28.1632 1.0000	38.0411 1.0000	28.3770 1.0000	38.8121 1.0000

Attack name	Gaussian noise 0.3 + JPEG Compression 90		Salt & pepper noise 0.3 + JPEG Compression 90	Rotation 2° + JPEG Compression 10	Scaling 0.5 + JPEG Compression 90
Avion	PSNR NCC	26.7857 1.0000	27.5880 1.0000	29.9711 1.0000	36.7074 1.0000
Jelly	PSNR NCC	27.5794 1.0000	28.1134 1.0000	29.2876 0.9995	39.4797 0.9996
Baboon	PSNR NCC	27.6385 1.0000	28.2293 1.0000	28.0350 1.0000	30.4527 0.9991
Sailboat	PSNR NCC	27.8243 1.0000	28.3414 1.0000	28.8169 1.0000	33.2983 1.0000
Female	PSNR NCC	27.7297 1.0000	28.1993 1.0000	30.1984 1.0000	42.5794 1.0000
Splash	PSNR NCC	28.0057 1.0000	28.3145 1.0000	30.0386 1.0000	38.5080 1.0000
Porthead	PSNR NCC	27.3668 1.0000	28.0204 1.0000	30.4698 1.0000	40.9663 1.0000

#### Robustness to geometrical attacks

Geometrical attacks often cause watermark detection to desynchronize. The two most common geometrical attacks are rotation and scaling. This experiment performs rotation-based attacks using nearest, bilinear, and bicubic interpolations. The test image undergoes rotation at 1°, 3°, 5°, 10°, and 50°, with the results shown in Table 3. For most angles, the NCC values become 1.0, and the BER values become 0, demonstrating that this approach is robust against rotation-based attacks. After that, the test image undergoes resizing using several methods, including scaling with factors of 0.25, 0.5, 2.0, and 4.0. Scaling attacks employ three interpolations: ‘Nearest,’ ‘Bilinear,’ and ‘Bicubic,’ with the results shown in Table 4. Most factors have ideal NCC values of 1.0 and ideal BER values of 0, suggesting complete robustness to scaling attacks.

#### Robustness to image processing attacks

The resistance of the suggested algorithm is assessed using standard image processing attacks, including filtering, noise addition, JPEG compression, unsharp masking (sharpening), and histogram equalization, on the test color image “House.” Filtering attacks were first conducted, as shown in Table 2, which presents the filtered images, PSNR values, and BER and NCC values for the retrieved watermark images. Most values of BER and NCC achieve the ideal values of 0 and 1.0, respectively, indicating a high resemblance between the original and extracted watermarks. These results confirm the suggested approach’s strong resilience against image-filtering attacks. The test color image was subjected to noise attacks utilizing salt-and-pepper and Gaussian noises. Table 5 presents the images after noise addition, their PSNR values, the extracted watermark images, and their accompanying BER and NCC values.

For Gaussian noise, all values of BER and NCC achieved the optimal values of 0 and 1.0, respectively. In the case of Salt-and-Pepper noise, three values reached the ideal BER and NCC values of 0 and 1.0. These results demonstrate that the suggested method exhibits strong resilience to noise attacks, ensuring the retrieved watermarks remain highly similar to the original ones. Subsequently, the test color image underwent JPEG compression attacks with quality factors of 5%, 10%, 50%, and 90%. Table 6 presents the images after compression, their PSNR values, and the corresponding BER and NCC values for the retrieved watermarks. Across all quality factors, the BER and NCC consistently achieved their ideal values of 0 and 1.0, respectively, with the retrieved watermarks being identical to the originals.

These results demonstrate the proposed method’s complete resistance to JPEG compression attacks. Lastly, the Effectiveness of Sharpening and histogram equalization attacks was evaluated, and their results, including PSNR values, attacked images, and retrieved watermark metrics (BER and NCC), are presented in Table 6. Translation attack outcomes are shown in Table 7, while cropping attack results are detailed in Table 9. Additionally, the effects of conventional combined attacks are summarized in Table 8. Across these scenarios, the values of BER and NCC predominantly align with the optimal values of 0 and 1.0, respectively. Moreover, the extracted watermarks are either highly close to or identical to the originals, underscoring the robustness of the proposed approach against these attacks.

### Security analysis

The security of the proposed watermarking scheme is significantly enhanced through a dual-chaotic encryption mechanism and zero-watermarking strategy. Initially, the watermark undergoes permutation based on chaotic sequences generated by the Lorenz system, which is highly sensitive to its initial conditions (x₀, y₀, z₀) and control parameters (σ, ρ, β, ∆t). These parameters act as the first secret key (SK1), introducing high sensitivity and unpredictability to the watermark position. In parallel, a second layer of encryption is applied using a Logistic Map, which produces a binary sequence based on the control parameter μ = 3.999. The watermark is then XORed with this sequence to form the final encrypted watermark. The combination of permutation and XOR-based masking ensures that even if part of the watermark is intercepted, it remains unrecoverable without knowledge of both chaotic keys.

To evaluate the resistance of the proposed scheme against brute-force attacks, the key space of the chaotic encryption mechanism is analyzed. The first key (SK1) is derived from six floating-point parameters in the Lorenz system. Assuming a computational precision of 10^15^, the number of possible combinations for SK1 is approximately:

$$\text{Key Space of the Lorenz system }= {\left({10}^{15}\right)}^{6}= {10}^{90}$$

The Logistic Map encryption introduces two additional parameters (initial value and control parameter), each with similar precision:

$$\text{Key Space of the Logistic Map }={\left({10}^{15}\right)}^{2}= {10}^{30}$$

Hence, the total key space of the system becomes:

$$\text{Total Key Space }= {10}^{90}\times {10}^{30}={10}^{120}\approx {2}^{398}$$

This vast key space far exceeds the 2^128^ benchmark commonly required for modern cryptographic security, making brute-force attacks computationally infeasible.

Additionally, the scheme follows a zero-watermarking model, where the host image is not modified directly. Instead, the watermark is logically bound to features extracted from the image (via VGG19 and LBP), further increasing the difficulty of tampering or forgery. Even if the image is stolen or altered, verification cannot be performed without access to the correct feature and encryption information. This layered approach, which combines spatial feature binding, dual chaotic encryption, and logical embedding, provides strong resistance against unauthorized extraction, reverse engineering, and key guessing, thereby fulfilling the requirements for robustness and security.

### Computational complexity analysis

The computational complexity of the proposed zero-watermarking scheme is analyzed step-by-step as follows:

1. LBP feature extraction
  For an input image of size $M \times N$, the LBP value is computed for each non-border pixel using a 3 × 3 sliding window.
  - Time Complexity*:* $$O(M \times N)$$
2. VGG19 feature extraction
  The image is passed through the VGG19 network up to the conv5_4 layer to extract deep semantic features. This step involves several convolution and pooling operations across multiple layers, and its cost is abstracted as *T*, representing the internal complexity of VGG19 up to the conv5_4 layer.
  - Time Complexity*:* $$O(T)$$
3. Feature fusion
  The extracted LBP and VGG19 features are flattened and concatenated into a single feature vector.
  - Time Complexity: $O(M \times N + P)$, where *P* is the size of the conv5_4 feature map.
4. Binarization
  The combined feature vector is binarized and reshaped into an $m \times n$ matrix.
  - Time Complexity: $$O(m \times n)$$
5. Chaotic Encryption using Lorenz and Logistic Map
  A chaotic permutation using the Lorenz system is applied (requires sorting), followed by XOR operations for watermark encryption.
  - Time Complexity: $$O(m \times n log(m \times n) + 3 \times m \times n)$$
6. Attack Simulation (e.g., filtering, noise, compression)
  Image attacks are applied over the host image, generally affecting the full $M \times N$ space.
  - Time Complexity: $$O(M \times N)$$
7. Watermark Extraction
  The same LBP and VGG19 extraction are repeated on the attacked image, followed by binarization and inverse permutation.
  - Time Complexity: $$O(M \times N + T + m \times n)$$
8. Quality Metric Computation (PSNR, BER, NCC)
  Metrics are computed over the m × n extracted and original watermark.
  - Time Complexity: $$O(3 \times m \times n)$$

Hence, summing the complexities of all steps, the total computational complexity of the proposed scheme is: $O(4MN + 2T + P + m n log(m n) + 8mn)$

To further support the analysis presented above, Table 16 presents a comparison between the proposed method and four recent state-of-the-art methods^5,8,10,43^, highlighting the overall computational cost in terms of Big-O notation. The results confirm that the proposed method requires less computational effort, making it more suitable for real-time processing and large-scale applications.

**Table 16:** Comparison of computational complexity between the proposed method and existing methods^5,8,10,43^.

Scheme	Total computational complexity
Proposed	$O(4MN + 2T + P + m n log(m n) + 8mn)$
^5^	O(n3)
^8^	$(O\left({M}^{2}\right)+O\left({M}^{2}\right))\approx O({M}^{2})$
^10^	$O({N}^{3})$
^43^	$O({L}^{3})$

### Wilcoxon signed-rank test comparison

Table 17 presents a statistical comparison between the proposed watermarking method and four existing techniques^13,44,45^, and^46^ under different types of attacks: Average filter, Median filter, Gaussian noise, JPEG compression, Rotation, and Scaling. Each row corresponds to a specific kind of attack, while each column represents the outcome of the Wilcoxon signed-rank test comparing the proposed method with one of the compared schemes.

**Table 17:** Statistical comparison using the Wilcoxon signed-rank testing under the considered attacks.

Attack	^13^	^44^	^45^	^46^
Average filter	Cannot reject ${H}_{0}$, with α = 0.05	Reject ${H}_{0}$, with α = 0.00222, *w*′ = 0 ≤ ${w}_{\alpha }^{*}$ = 13	Reject ${H}_{0}$, with α = 0.00438, *w*′ = 1 ≤ ${w}_{\alpha }^{*}$ = 10	Reject ${H}_{0}$, with α = 0.00222, *w*′ = 0 ≤ ${w}_{\alpha }^{*}$ = 13
Median filter	Reject ${H}_{0}$, with α = 0.02852, *w*′ = 6 ≤ ${w}_{\alpha }^{*}$ = 8	Reject ${H}_{0}$, with α = 0.00222, *w*′ = 0 ≤ ${w}_{\alpha }^{*}$ = 13	Reject ${H}_{0}$, with α = 0.00438, *w*′ = 1 ≤ ${w}_{\alpha }^{*}$ = 10	Reject ${H}_{0}$, with α = 0.00222, *w*′ = 0 ≤ ${w}_{\alpha }^{*}$ = 13
Gaussian noise	Reject ${H}_{0}$, with α = 0.0198, *w*′ = 19 ≤ ${w}_{\alpha }^{*}$ = 25	Reject ${H}_{0}$, with α = 0.00148, *w*′ = 0 ≤ ${w}_{\alpha }^{*}$ = 17	Reject ${H}_{0}$, with α = 0.0164, *w*′ = 6 ≤ ${w}_{\alpha }^{*}$ = 10	Reject ${H}_{0}$, with α = 0.00044, *w*′ = 0 ≤ ${w}_{\alpha }^{*}$ = 29
JPEG compression	Reject ${H}_{0}$, with α = 0.01878, *w*′ = 9 ≤ ${w}_{\alpha }^{*}$ = 13	Reject ${H}_{0}$, with α = 0.00222, *w*′ = 0 ≤ ${w}_{\alpha }^{*}$ = 13	Reject $H_{0}$, with α = 0.00512, *w*′ = 0 ≤ $w_{\alpha }^{*}$. = 8	Reject $H_{0}$, with α = 0.00222, *w*′ = 0 ≤ $w_{\alpha }^{*}$ = 13
Rotation	Reject $H_{0}$, with α = 0.03236, *w*′ = 6.5 ≤ $w_{\alpha }^{*}$. = 8	Reject $H_{0}$. , with α = 0.00222, *w*′ = 0 ≤ $w_{\alpha }^{*}$ = 13	Reject $H_{0}$, with α = 0.00222, *w*′ = 0 ≤ $w_{\alpha }^{*}$ = 13	Reject $H_{0}$, with α = 0.00222, *w*′ = 0 ≤ $w_{\alpha }^{*}$ = 13
Scaling	Cannot reject $H_{0}$, with α = 0.65994	Reject $H_{0}$, with α = 0.00044, *w*′ = 0 ≤ $w_{\alpha }^{*}$ = 29	Cannot reject $H_{0}$, with α = 0.50926	Reject $H_{0}$, with α = 0.0477, *w*′ = 21 ≤ $w_{\alpha }^{*}$ = 21

The Wilcoxon signed-rank test was applied independently for each attack type at a significance level of α = 0.05, indicating a 95% confidence level for the statistical test. The test is non-parametric and is commonly used to assess whether there is a statistically significant difference between two related samples. For each comparison, the null hypothesis (${H}_{0}$) states that there is no statistically significant difference in performance between the proposed and the compared method. A result of rejecting the null hypothesis H0 indicates that the proposed method achieves significantly different results. In contrast, a result of not rejecting the null ${H}_{0}$ suggests that no meaningful statistical difference could be identified under the given test conditions. For the statistically significant cases, the test also compares the sum of negative ranks (*w*′) with the corresponding critical value (${w}_{{\alpha }}^{*}$).

If *w*′ ≤ ${w}_{{\alpha }}^{*}$, this provides further evidence that the proposed method outperforms the compared one under the given attack. As observed in the table, the proposed method achieves statistically significant improvements over all the compared methods in most attacks, particularly under Median filter, Gaussian noise, JPEG compression, and Rotation attacks. However, under the Scaling and Average filter attacks, the difference is not statistically significant against methods^13^ and^45^, which suggests performance parity in those specific scenarios. Overall, this statistical validation confirms the robustness and superiority of the proposed scheme under a wide range of attack conditions.

### Comparing robustness to previous works

**Fig. 7:**
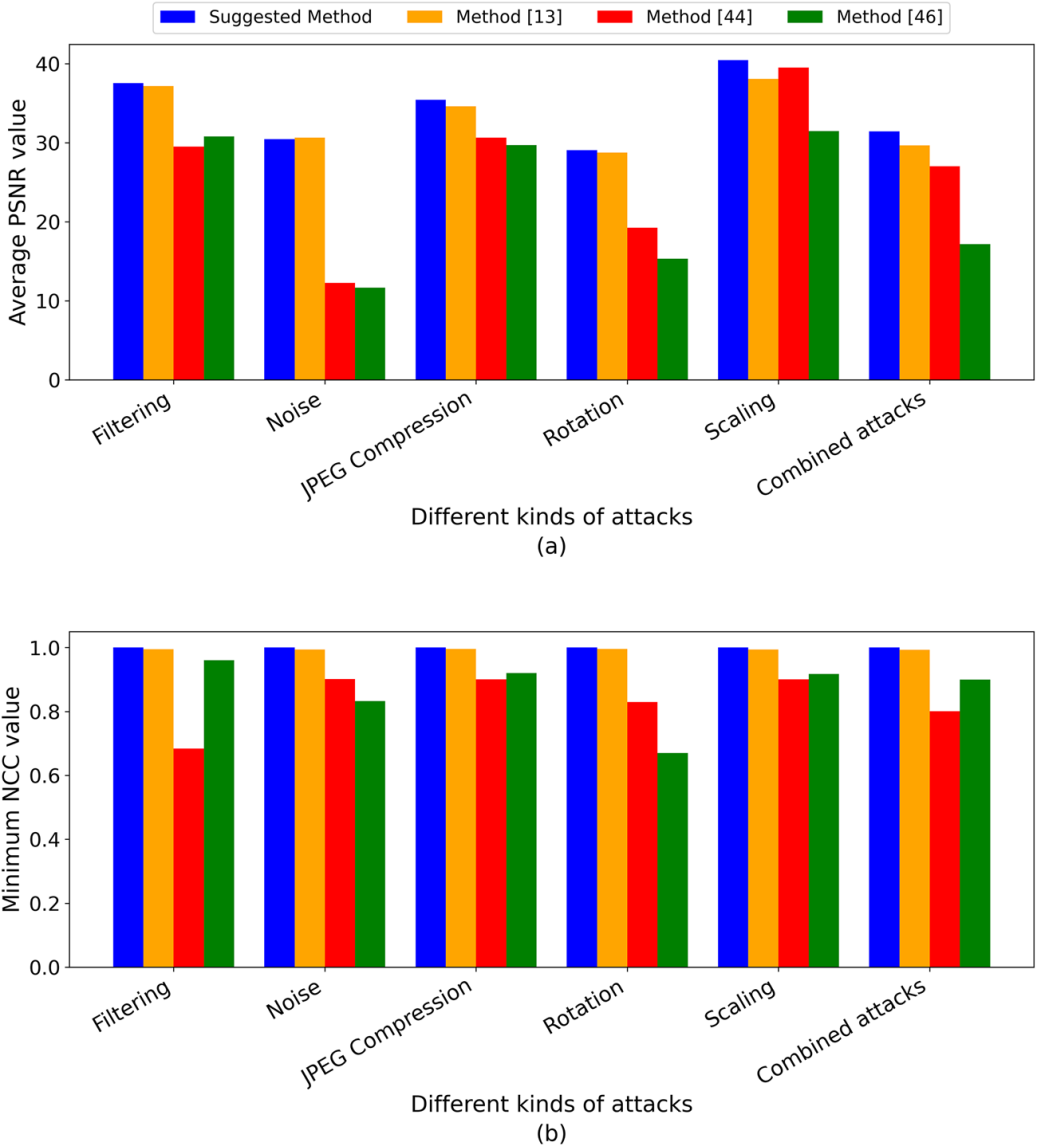
Comparison of the suggested algorithm with methods^13,44^, and^46^ under various attacks: (**a**) Average PSNR values, and (**b**) Minimum NCC values.

To comprehensively assess the efficiency of the suggested method, we performed two comparisons. In the first comparison, various attacks were applied to seven standard color images with parameters detailed in Table 1. The average PSNR values are then calculated, and the lowest NCC values are chosen, as presented in Tables 10, 11, 12, 13, 14, and 15. The summarized results are provided in Table 18 for clarity and are further illustrated in Fig. 7. Additionally, the results are compared with those from zero-watermarking methods in^13,44^, and^46^. Table 18 and Fig. 7a show that the proposed algorithm’s PSNR values are higher, indicating superior image resolution compared to the three methods. Similarly, Fig. 7b and Table 18 show that the values of NCC of the proposed method under various attacks are nearer to the ideal value of 1 and outperform those of the methods in^13,44^, and^46^. These findings highlight the resilience of the suggested approach in resisting different image attacks, surpassing the performance of zero-watermarking methods^13,44^, and^46^.

**Table 18:** Average PSNR and minimum NCC values under various attacks.

Type of attack		The average PSNR value			The lowest NCC value			
Suggested method		^13^	^44^	^46^	Suggested Method	^13^	^44^	^46^
Filtering (Average, Median, Winner, Gaussian)	37.5523	37.1699	29.5269	30.811	0.9982	0.9949	0.6841	0.96
Noise (Gaussian, Salt and Pepper)	30.4456	30.6423	12.2624	11.6486	0.9974	0.9942	0.9014	0.8321
JPEG Compression (5, 10, 20, 30, 40, 50, 60, 70, 80, 90)	35.4442	34.6193	30.6377	29.7121	0.9959	0.9953	0.9002	0.92
Rotation (Nearest, Bilinear, Bicubic)	29.0768	28.7528	19.2357	15.3149	0.9986	0.9961	0.8292	0.67
Scaling (Nearest, Bilinear, Bicubic)	40.4647	38.0713	39.5427	31.4808	0.9973	0.9937	0.9002	0.9169
Conventional Combined attacks	31.4317	29.6665	27.0336	17.1641	0.9986	0.9929	0.8011	0.90

For the second comparison, the resistance of the suggested algorithm was thoroughly evaluated against several zero-watermarking techniques reported in previous studies^13,43,44,47,48,45,46^. This evaluation encompassed a diverse range of attacks to ensure comprehensive testing of the algorithm’s capabilities. The tested attacks included JPEG compression with quality factors of 10%, 30%, 50%, 70%, and 90%; rotation at angles of 3° and 5°; and scaling with a factor of 0.5. Additionally, noise attacks were conducted, including salt-and-pepper noise with parameters of 0.01 and 0.03 and Gaussian noise with 0.1, 0.3, 0.5, and 0.01. Filtering attacks were also applied, involving average filtering and Median filtering with window sizes of 3 × 3, 5 × 5, and 7 × 7, as well as the sharpening attack. As presented in Table 19, the experimental results demonstrate the superior performance of the proposed algorithm, which consistently achieves bit error rate (BER) values close to the optimal value of 0. This outcome reflects the algorithm’s significantly enhanced resilience to various attacks, surpassing the robustness of existing zero-watermarking methods.

**Table 19:** Comparison of BER values between the proposed and existing methods^13,43,44,47,48,45,46^ under various attacks.

Attacks	BER							
	Suggested algorithm	^13^	^43^	^44^	^47^	^48^	^45^	^46^
Gaussian noise (0.1)	0	0.0002	0.0034	0.001	0.0010	0.01	0.0082	–
Gaussian noise (0.3)	0	0	0.0084	0.0026	0.0010	0.0106	0.0092	–
Gaussian noise (0.5)	0	0.0002	0.0093	0.0036	0.0010	0.0121	0.0102	–
Gaussian noise (0.01)	0	0	0.0323	0.0091	–	0.0296	0.0421	0.0172
Salt & Pepper noise (0.01)	0	0	0.0125	0.0091	–	0.0421	0.0926	0.0091
Salt & Pepper noise (0.03)	0.0005	–	0.0290	0.0124	0.0010	0.0542	0.116	–
JPEG compression (F = 10)	0	0.0002	0.0253	0.0041	0	0.1231	0.4844	–
JPEG compression (F = 30)	0	0	0.0135	0.0037	–	0.0983	0.4063	0.0131
JPEG compression (F = 50)	0	0	0.0111	0.0021	0	0.0849	0.3438	0.0099
JPEG compression (F = 70)	0	0	0.0039	0.0007	0.0005	0.0183	0.0713	0.0058
JPEG compression (F = 90)	0	0	0.0018	0.0002	0.0002	0.0073	0.0213	–
Average filter (3 × 3)	0	0	0.0099	0.202	0.0001	0.0029	0.099	0.0079
Average filter (5 × 5)	0	0.0002	0.0181	0.0361	0.0001	0.298	0.0455	0.0157
Average filter (7 × 7)	0	0.0009	0.0245	0.0618	0.0001	0.2919	0.0630	–
Median filter (3 × 3)	0.0005	0.0002	0.0068	0.001	0	0.0039	0.0442	0.0064
Median filter (5 × 5)	0	0	0.0135	0.009	0.0007	0.02866	0.0505	0.0099
Median filter (7 × 7)	0	0.0002	0.0195	0.011	0.0001	0.02910	0.0984	–
Rotation (3°)	0.0005	0	–	0.00959	0.0002	0	–	0.1333
Rotation (5°)	0	0	–	0.02982	0.0002	0	–	0.1858
Scaling (0.5)	0	0	–	0.00659	0.0002	0	–	0.0071
Sharpening	0	0	0.0007	0	0.0001	0.00018	0.0009	–

## Conclusion

This paper proposes a reliable zero-watermarking technique for color images, which combines Local Binary Pattern (LBP) and deep features from the CONV5-4 layer of VGG19 to enhance resilience and security. Frequency domain transformations, such as those using the DWT and DCT, isolate critical features. At the same time, chaotic encryption relies on the Lorenz system, and the Logistic map secures the watermark by scrambling the feature matrix and the watermark image. The watermark is embedded by modifying high-frequency coefficients, ensuring imperceptibility. The algorithm is resilient to attacks such as scaling, compression, noise addition, filtering, and rotation, while maintaining excellent image quality and robust ownership verification. Experimental results demonstrate that the proposed approach surpasses conventional zero-watermarking algorithms in terms of resilience, security, and image preservation, effectively preserving the copyright of color images. In the future, the proposed technique will be expanded to video watermarking to enhance its security and resilience, thereby meeting the demands of dynamic multimedia content. Furthermore, its use in e-healthcare, telemedicine, stereoscopic imaging, and real-time acquired pictures will be investigated.

## Data Availability

The data supporting this study’s findings are available from the corresponding author upon request.
